# Artificial intelligence based image quality enhancement in liver MRI: a quantitative and qualitative evaluation

**DOI:** 10.1007/s11547-022-01539-9

**Published:** 2022-09-07

**Authors:** Marta Zerunian, Francesco Pucciarelli, Damiano Caruso, Michela Polici, Benedetta Masci, Gisella Guido, Domenico De Santis, Daniele Polverari, Daniele Principessa, Antonella Benvenga, Elsa Iannicelli, Andrea Laghi

**Affiliations:** Department of Medical Surgical Sciences and Translational Medicine, Sapienza University of Rome – Sant’Andrea University Hospital, Via di Grottarossa, 1035-1039, 00189 Rome, Italy

**Keywords:** Artificial intelligence, Image quality, Scanning time, Sequences optimization

## Abstract

**Purpose:**

To compare liver MRI with AIR Recon Deep Learning™(ARDL) algorithm applied and turned-off (NON-DL) with conventional high-resolution acquisition (NAÏVE) sequences, in terms of quantitative and qualitative image analysis and scanning time.

**Material and methods:**

This prospective study included fifty consecutive volunteers (31 female, mean age 55.5 ± 20 years) from September to November 2021. 1.5 T MRI was performed and included three sets of images: axial single-shot fast spin-echo (SSFSE) T2 images, diffusion-weighted images(DWI) and apparent diffusion coefficient(ADC) maps acquired with both ARDL and NAÏVE protocol; the NON-DL images, were also assessed. Two radiologists in consensus drew fixed regions of interest in liver parenchyma to calculate signal-to-noise-ratio (SNR) and contrast to-noise-ratio (CNR). Subjective image quality was assessed by two other radiologists independently with a five-point Likert scale. Acquisition time was recorded.

**Results:**

SSFSE T2 objective analysis showed higher SNR and CNR for ARDL vs NAÏVE, ARDL vs NON-DL(all *P* < 0.013). Regarding DWI, no differences were found for SNR with ARDL vs NAÏVE and, ARDL vs NON-DL (all *P* > 0.2517).CNR was higher for ARDL vs NON-DL(*P* = 0.0170), whereas no differences were found between ARDL and NAÏVE(*P* = 1). No differences were observed for all three comparisons, in terms of SNR and CNR, for ADC maps (all *P* > 0.32).

Qualitative analysis for all sequences showed better overall image quality for ARDL with lower truncation artifacts, higher sharpness and contrast (all *P* < 0.0070) with excellent inter-rater agreement (*k* ≥ 0.8143). Acquisition time was lower in ARDL sequences compared to NAÏVE (SSFSE T2 = 19.08 ± 2.5 s vs. 24.1 ± 2 s and DWI = 207.3 ± 54 s vs. 513.6 ± 98.6 s, all *P* < 0.0001).

**Conclusion:**

ARDL applied on upper abdomen showed overall better image quality and reduced scanning time compared with NAÏVE protocol.

## Introduction

Medical imaging plays a central role in modern medicine and technical improvements are fundamental to achieve the best diagnostic performances with the most efficient imaging protocol. In recent years Artificial Intelligence (AI) allowed huge improvements in different aspects such as image acquisition/reconstruction, image post-processing, image analysis, image storage, and integration of complex data for medical decision-making process [1–7].

Focusing on image acquisition, several in-house AI and deep-learning (DL) tools have been developed to improve image quality and their application since now has been mainly in research field [8].

In September 2020 one of the first AI-derived tool has been approved for clinical use for all anatomies at 1.5 T, the AIR™ Recon DL (ARDL), GE Healthcare, Waukesha, WI [9]. Unlike post-processing-based approaches that might alter image detail, this method is a deep learning-based reconstruction model applied directly on k-space-based raw data for maximum image quality, by preserving non-DL data acquired. In addition, to improve signal-to-noise ratio (SNR), this technology has a unique intelligent ringing suppression that preserves fine image details, helping address two common delicate aspects for radiologists and technologists that are image noise and ringing. In addition, with ARDL is possible to obtain simultaneously a dataset, called NON-DL, resulted from the same MRI parameters set without the intervention of ARDL on the sequence.

Until now, MRI acquisition has been a compromise between image quality and scan time; in fact, to achieve high-quality images with improved SNR and/or spatial resolution, necessitated long scan times. On the contrary, shorter scan acquisition time enables to improve patient comfort and productivity, with reduced image quality. With new DL algorithms, the possibility to achieve good image quality in reduced acquisition time is becoming a reality. Moreover, it is important to compare the new algorithms not only with standard protocol but also with high-quality protocol, more similar to the ground truth.

Up to now, a few studies has assessed the impact of AI on MRI acquisition in clinical setting and in particular on upper abdomen [10–13].

The aim of the study is to compare ARDL-protocol, NON-DL dataset and high-resolution NAÏVE protocol applied to upper abdomen district, in terms of quantitative and qualitative image analysis and acquisition time.

## Methods

### Study design and patient population

This prospective single-center study included 50 volunteers from September 2021 to November 2021, in accordance with local IRB, and informed consent was signed by all participants.

All volunteers underwent upper abdomen MRI scan and both protocols (ARDL and NAÏVE) were performed; details of the two protocols and three datasets obtained are described below in *Deep-learning algorithm and Imaging protocol* sections Volunteers with contraindications to perform MRI (e.g. non-compatible MRI devices, claustrophobia), and volunteers who underwent upper abdomen surgery were excluded; also MRI acquisitions with severe motion artefacts or magnetic susceptibility artefacts related to abdominal metallic devices were not included in the analysis.

### Deep-learning algorithm

The specific DL algorithm applied during image acquisition is the AIR™ Recon DL, GE Healthcare, Waukesha, WI. It consists of a feed-forward deep convolutional neural network (CNN) that enables to reconstruct images with higher SNR, reduced truncation artifacts, and higher spatial resolution [14]. This innovative CNN works directly integrated with the standard reconstruction pipeline, on the raw data obtained during MRI examination. A supervised learning approach with over 4.4 million parameters in over 10.000 kernels has been used to train this CNN.

AIR™ Recon DL works by accepting raw, unfiltered input and to provide improved output in terms of SNR and image artefacts; another important aspect is the possibility to set the range of applicability of the CNN before the acquisition to generate images with a different CNN application strength (Low, Medium and High).

From each acquisition are also obtained two different dataset available simultaneously during the scanning, without different reconstruction time. In particular, one dataset is reconstructed with ARDL applied and, the other one, defined as NON-DL, resulted from the same parameters set without the ARDL reconstruction on k-space. It is important to underline that NON-DL dataset returns the conventional reconstruction with non-optimized parameters so, without the ARDL reconstruction. As a result, images will appear noisier, due to the lack of the usual parameters that are set to balance image quality and acquisition time.

An image artefact that the AIR™ Recon DL is trained to recognize and reduce is the truncation (Gibbs) artefact, usually present near structures with transition between regions of high and low signal intensity such as spinal cord and cerebral-spinal fluid or liver parenchyma and abdominal fat [15]. The CNN is able to recognize Gibbs artefact in proximity of sharp edges and reduce consistently the ringing artefact to improve image sharpness. As a result, ARDL tool has the possibility to acquire MRI images with less compromise than usually in terms of MRI parameters (e.g. reduced NEX, thickness) and acquisition time.

### Imaging protocol

Each acquisition has been performed on the same scanner (1.5 T Signa Voyager, GE Healthcare, Waukesha, WI) with the same protocol. All scans have been performed in supine position, with a dedicated 16-channel highly flexible Adaptive Image Receiver (AIR, GE Healthcare, Waukesha, WI) coil and, no contrast medium administration. Field of View (FOV) will include upper abdomen coverage from pulmonary bases to the upper margin of iliac bones, with the same number of slices for both protocols.

Acquisition protocol includes high-quality NAÏVE protocol and ARDL protocol, the latter with two reconstruction dataset (ARDL and NON-DL datasets) as described above.

Acquisition protocol included axial T2 single-shot fast spin-echo (SSFSE) sequences acquired with breath-hold technique for both ARDL protocol (TR: 412 ms, TE:92 ms, nex: 1, slice thickness: 4 mm matrix: 384X288, FOV: 430 × 430 mm, Recon DL strength: High) and NAÏVE protocol (TR: 502 ms, TE:89 ms, nex: 1, slice thickness: 4 mm, spacing: 0.5 matrix: 232X200, FOV: 430 × 430 mm). Then, axial DWI will be acquired with navigator-triggered technique for both ARDL protocol (TR: 6000 ms, TE:61 ms, slice thickness: 4 mm, spacing: 0.5, matrix: 128X128, FOV: 430 × 430 mm, b values acquired: b50, nex: 2; b400, nex:2, b1000, nex: 4, Recon DL strength: Medium) and NAÏVE protocol (TR: 14811 ms, TE:57 ms, slice thickness: 4 mm, spacing: 0.5, matrix: 128X128, FOV: 430 × 430 mm, b values acquired: b50, nex: 2; b400, nex:8, b1000, nex: 12). ARDL reconstruction has been chosen among Low, Medium and High options after a pilot protocol consisted in five volunteers scanning with the various DL strength applied. The obtained images were assessed by three expert readers that chose in consensus the DL strength for both sequences. ADC maps obtained from DWI of both protocols have been also analyzed. During ARDL-protocol also native images (named NON-DL as abovementioned) were obtained and included in the analysis. Acquisition time was also recorded for each sequence.

### Quantitative Image analysis

**Fig. 1:**
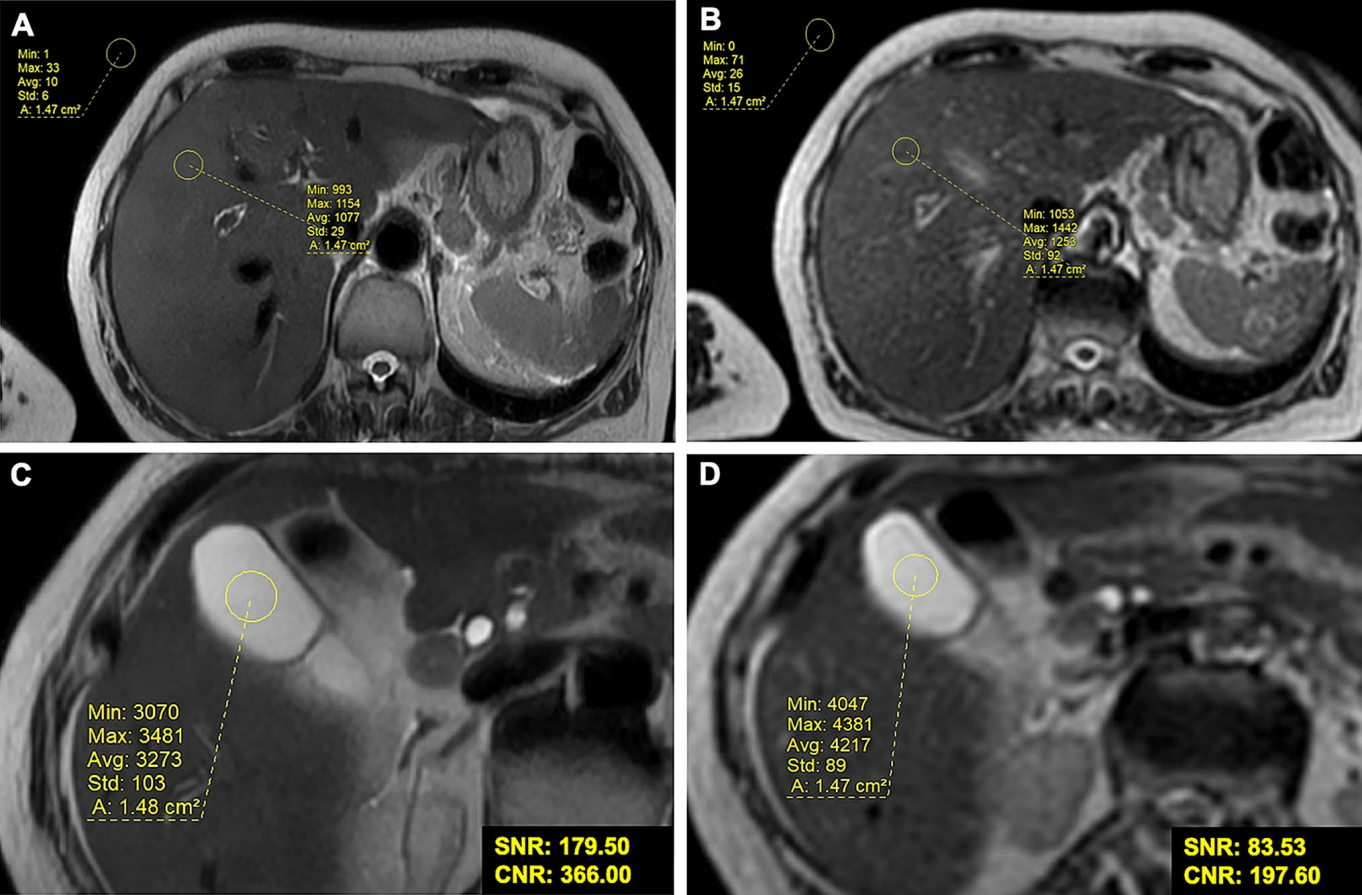
ROIs placement on MRI scan for quantitative analysis. Unenhanced axial SSFSE T2 images of a 52-years-old male with one of the three ROIs placed on the V hepatic segment liver, one ROI on the background and one in the gallbladder with ARDL **a**, **c** NAÏVE **b**, **d** showing significant differences in SNR and CNR between ARDL and NAÏVE images

A total of three dataset have been obtain for each sequence as follows: ARDL dataset, NAÏVE dataset and NON-DL dataset, each for T2 SSFSE sequence, DWI and for ADC maps. Each sequence was anonymized in order to avoid bias. Two readers (B.M. and M.Z., with 3 and 8 years of experience in abdominal MRI imaging respectively) assessed objective image quality in consensus and simultaneously. In particular, to quantify signal-to-noise ratio (SNR) and contrast-to-noise ratio (CNR) three circular regions of interest (area 1cm^2^) were placed in the liver parenchyma at the V, VI and VIII segments, by avoiding vessels and biliary tree, and liver edge, and then, the averaged measurements of the three was reported. In addition and a single region of interest (ROI) in the background and in the gallbladder were placed. Explicatory single slice of the ROIs placement is provided in Fig. 1.

SNR and CNR were calculated with the following formulas modified by previous study [12]:

$$\begin{aligned} {\text{SNR}} & = {\text{S}}_{{{\text{liver}}}} /{\text{SD}}_{{{\text{background}}}} \\ {\text{CNR}} & = |{\text{S}}_{{\text{gall - bladder}}} {-}{\text{S}}_{{{\text{liver}}}} |/{\text{ SD}}_{{{\text{background}}}} \\ \end{aligned}$$

where S represents mean signal intensity while SD represents signal intensity standard deviation.

### Qualitative image analysis

Two readers (M.P. and G.G.) with 6 and 7 years of experience in abdominal MRI imaging respectively, independently assessed subjective image quality. Subjective image quality has been assessed with a five-point Likert scale considering: (a) upper abdomen parenchyma edge sharpness: 1 = poor; 2 = mild; 3 = moderate; 4 = good; 5 = very good.

**Fig. 2:**
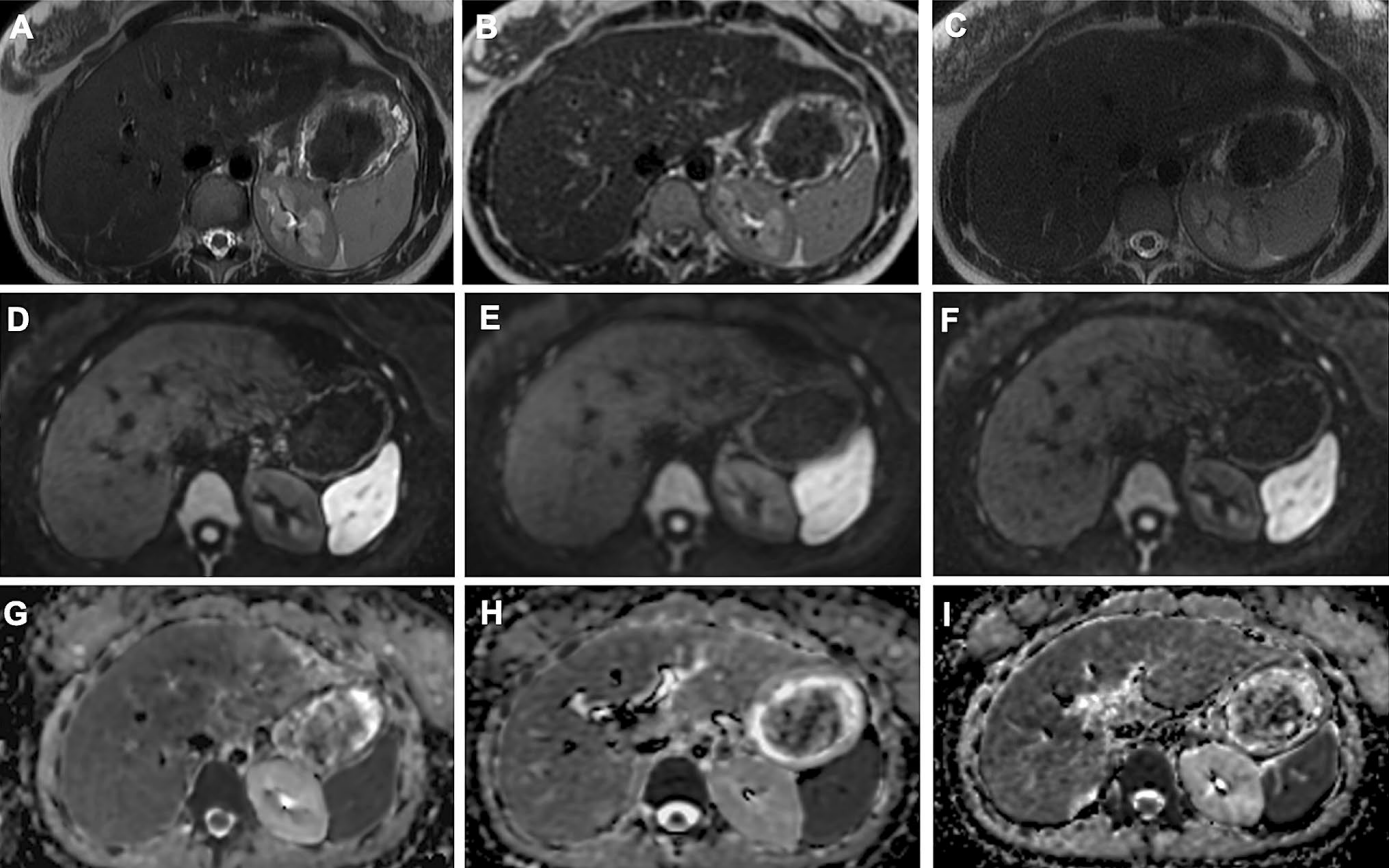
Comparison of ARDL, NAÏVE, NON-DL dataset on SSFSE T2, DWI and ADC. Female, 28-years old underwent unenhanced upper-abdomen MRI. Axial images showing SSFSE T2 sequences with ARDL **a**, NAÏVE **b**, NON-DL **c** dataset respectively; DWI sequences with ARDL **d**, NAÏVE **e**, NON-DL **f**; ADC maps with ARDL **g**, NAÏVE **h** and NON-DL **i**. Anonymized datasets were obtained to perform qualitative image analysis with 5-point Likert scale assessing Sharpness, Contrast, Truncation artefacts, Motion artifacts and Overall image quality. ARDL dataset showed higher image quality in all datasets in terms of overall image quality compared to NAÏVE and NON-DL dataset.

(b) contrast: 1 = insufficient; 2 = mild; 3 = moderate; 4 = good; 5 = excellent.

(c) truncation artifacts: 1 = severe (hindering diagnosis); 2 = acceptable; 3 = moderate; 4 = mild; 5 = absence of artifacts.

(d) motion artifacts: 1 = severe (hindering diagnosis); 2 = acceptable; 3 = moderate; 4 = mild; 5 = absence of artifacts.

(e) overall image quality (the four factors above added together): 1 = unacceptable; 2 = poor; 3 = moderate; 4 = good; 5 = excellent. Example of the qualitative dataset analyzed is provided in Fig. 2.

### Statistical analysis

Continuous variables were compared using the paired-samples t-test or the Wilcoxon test according to the normality of data distribution priorly assessed with Kolmogorov–Smirnov Test. Multiple tests were assessed by One way repeated measures ANOVA test for the SNR and the CNRand, for the qualitative analysis, with Bonferroni correction of the *P* values. The t-test or Wilcoxon signed-rank test will be adopted to compare the acquisition times between ARDL and NAÏVE MRI protocols.

The intraclass correlation coefficient (ICC) will be used to investigate the inter-observer agreement of qualitative values (0.21–0.40, fair; 0.41–0.60, moderate; 0.61–0.80, good; 0.81–1.00, excellent).

A two-sided P value < 0.05 was considered statistically significant.

All statistical analyses will be performed using SPSS (21.0; SPSS, Chicago, IL, USA) and MedCalc version 12.7.2 (MedCalc Software, Ostend, Belgium).

## Results

### Patient population

From an initial population of 57 volunteers, 5 were excluded for ferromagnetic artifacts due to surgery, one for incomplete MRI protocol due to claustrophobia and one due to several motion artifact. The final population included 50 volunteers (19 male, 31 female), mean age 55.5 ± 20 years old.

### Quantitative analysis

Axial SSFSE T2 quantitative analysis showed significantly higher SNR for ARDL protocol (181.40 ± 135.09) compared with NAÏVE protocol (109.79 ± 108.98, *P* = 0.0012), and NON-DL dataset (18.08 ± 12.26, *P* < 0.0001) and for NAÏVE protocol compared to NON-DL acquisitions (109.79 ± 108.98 vs. 18.08 ± 12.26, *P* < 0.0001)*.*

Axial SSFSE T2 CNR analysis showed significantly higher values for ARLD dataset (674.76 ± 453.82) compared with NAÏVE group (457.29 ± 449.25, *P* = 0.013), for ARDL compared with NON-DL protocol (68.66 ± 39.06, *P* < 0.0001) and for NAÏVE compared with NON-DL datasets (457.29 ± 449.25 vs. 68.66 ± 39.06, *P* < 0.0001).

Axial DWI sequences showed no significant differences for SNR between ARDL and NAÏVE protocols (181.15 ± 134.93 vs. 216.41 ± 157.47, *P* = 0.7231), ARDL and NON-DL datasets (181.15 ± 134.93 vs. 161.65 ± 155.87, *P* = 1) and NAÏVE and NON-DL sequences (261.41 ± 157.47vs. 161.65 ± 155.87, *P* = 0.2517).

Axial DWI sequences CNR showed no significant differences, between ARDL and NAÏVE protocol (92.58 ± 84.32 vs. 93.06 ± 83.50, *P* = 1); on the contrary, significant higher CNR was observed for ARDL protocol compared with NON-DL dataset (92.58 ± 84.32 vs. 54.36 ± 48.90, *P* = 0.0170) and NAÏVE protocol versus NON-DL one (93.06 ± 83.50 vs. 54.36 ± 48.90, *P* = 0.0177).

ADC maps SNR showed no significant differences between ARDL and NAÏVE protocols (3.97 ± 2.27 vs. 4.16 ± 1.60, *P* = 1), ARDL and NON-DL datasets (3.97 ± 2.27 vs. 3.85 ± 1.66, *P* = 0.3366) and NAÏVE and NON-DL maps (4.16 ± 1.60 vs. 3.85 ± 1.66, *P* = 0.5179).

ADC maps CNR showed no significant differences, for ARDL protocol and NAÏVE protocols (7.97 ± 5.34 vs. 7.19 ± 3.89, *P* = 1), ARDL protocol compared with NON-DL group (7.97 ± 5.34 vs. 7.55 ± 5.45, *P* = 0.32*)* and NAÏVE protocol in comparison with NON-DL dataset (7.19 ± 3.89 vs. 7.55 ± 5.45, *P* = 0.5733). All results are listed in Table 1.

**Table 1:** Results of quantitative analysis in terms of mean values ± deviation standard of signal-to-noise ratio (SNR) and contrast-to-noise ratio (CNR) in axial SSFE T2 and DWI sequences and ADC maps between ARDL and NAÏVE images, ARDL and NON-DL images and NAÏVE and NON-DL images

	ARDL	NAÏVE	NON-DL	ARDL vs NAÏVE (*P* value)	ARDL vs NON-DL (*P* value)	NAÏVE vs NON-DL (*P* value)
SSFE T2						
*SNR*	181.40 ± 135.09	109.79 ± 108.98	18.08 ± 12.26	**0.0012**	** < 0.0001**	** < 0.0001**
*CNR*	674.76 ± 453.82	457.29 ± 449.25	68.66 ± 39.06	**0.013**	** < 0.0001**	** < 0.0001**
DWI						
*SNR*	181.15 ± 134.93	216.41 ± 157.47	161.65 ± 155.87	0.7231	1	0.2517
*CNR*	92.58 ± 84.32	93.06 ± 83.50	54.36 ± 48.90	1	**0.0170**	**0.0177**
ADC						
*SNR*	3.97 ± 2.27	4.16 ± 1.60	3.85 ± 1.66	1	0.3366	0.5179
*CNR*	7.97 ± 5.34	7.19 ± 3.89	7.55 ± 5.45	1	0.32	0.5733

Significant ***P*** values are reported in bold

### Qualitative analysis

Qualitative image analysis showed higher significant results for the characteristics assessed (sharpness, truncation, contrast, motion) for SSFSE T2, DWI and ADC with ARDL protocol compared with NAÏVE protocol, and for ARDL protocol vs NON-DL sequences (all *P* < 0.05). Non-significant results were observed for NAÏVE protocol compared with NON-DL sequences (all *P* > 0.05) except for truncation artifact of DWI sequence (3.48 ± 0.83 vs 2.92 ± 1.02, *P* = 0.0146).

Overall image quality in SSFSE T2 resulted higher for ARDL protocol compared with NAÏVE protocol (4.94 ± 0.23 vs. 3.26 ± 0.75, *P* < 0.0001) and, ARDL compared with NON-DL dataset (4.94 ± 0.23 vs. 3.28 ± 0.60, *P* < 0.0001); non-significant differences were observed for NAÏVE protocol in comparison with NON-DL dataset (3.26 ± 0.75 vs. 3.28 ± 0.60, *P* = 1).

Overall image quality for DWI sequences resulted higher in ARDL protocol compared with NAÏVE one (4.22 ± 0.81 vs. 2.92 ± 0.85, *P* < 0.0001), and for ARDL compared with NON-DL datasets (4.22 ± 0.81 vs. 2.90 ± 0.73, *P* < 0.0001); non-significant differences were observed for NAÏVE protocol in comparison with NON-DL dataset (2.92 ± 0.85 vs. 2.90 ± 0.73, *P* = 1).

Overall image quality for ADC maps resulted higher in ARDL protocol compared with NAÏVE one (4.17 ± 0.77 vs. 3.07 ± 0.66, *P* < 0.0001), for ARDL compared with NON-DL datasets (4.17 ± 0.77 vs. 2.46 ± 0.79, *P* < 0.0001) and, for NAÏVE protocol in comparison with NON-DL dataset (3.07 ± 0.66 vs. 2.46 ± 0.79, *P* = 0.0070). Detailed results for all the qualitative analyses are listed in Table 2.

**Table 2:** Results of qualitative analysis, expressed as mean ± deviation standard, in axial SSFE T2 and DWI sequences and ADC maps between ARDL and NAÏVE images, ARDL and NON-DL images and NAÏVE dataset compared to NON-DL dataset

	ARDL	NAÏVE	NON-DL	ARDL vs NAÏVE *P* value)	ARDL vs NON-DL (*P* value)	NAÏVE vs NON-DL (*P* value)
SSFSE T2						
*Sharpness*	4.88 ± 0.32	3.1 ± 0.99	2.84 ± 0.65	** < 0.0001**	** < 0.0001**	0.0794
*Contrast*	4.9 ± 0.30	2.94 ± 0.91	2.82 ± 0.62	** < 0.0001**	** < 0.0001**	1
*Truncation artefacts*	4.78 ± 0.41	3.96 ± 0.80	3.6 ± 1.03	** < 0.0001**	** < 0.0001**	0.1371
*Motion artefacts*	4.86 ± 0.40	3.92 ± 0.77	3.72 ± 0.88	**0.0063**	** < 0.0001**	0.4523
*Overall*	4.94 ± 0.23	3.26 ± 0.75	3.28 ± 0.60	** < 0.0001**	** < 0.0001**	1
DWI						
*Sharpness*	4.16 ± 0.73	2.86 ± 0.92	2.72 ± 0.75	** < 0.00001**	** < 0.0001**	1
*Contrast*	4.14 ± 0.72	2.92 ± 0.92	2.66 ± 0.79	** < 0.00001**	** < 0.0001**	0.2542
*Truncation artefacts*	3.9 ± 0.97	3.48 ± 0.83	2.92 ± 1.02	**0.05**	** < 0.0001**	**0.0146**
*Motion artefacts*	4.18 ± 0.86	3.52 ± 1.01	3.22 ± 0.88	**0.0001**	** < 0.0001**	0.2242
*Overall*	4.22 ± 0.81	2.92 ± 0.85	2.90 ± 0.73	** < 0.0001**	** < 0.0001**	1
ADC						
*Sharpness*	4.17 ± 0.77	2.46 ± 0.83	2.53 ± 0.79	** < 0.0001**	** < 0.0001**	1
*Contrast*	4.14 ± 0.80	2.32 ± 0.66	2.53 ± 0.92	** < 0.0001**	** < 0.0001**	0.7388
*Truncation artefacts*	3.78 ± 0.91	2.53 ± 1.45	2.78 ± 1.28	** < 0.0001**	**0.0001**	0.2093
*Motion artefacts*	3.85 ± 0.93	2.67 ± 1.33	2.82 ± 1.18	** < 0.00001**	**0.0002**	0.9786
*Overall*	4.17 ± 0.77	3.07 ± 0.66	2.46 ± 0.79	** < 0.0001**	** < 0.0001**	**0.0070**

Significant *P* values are expressed in bold

Interrater agreement regarding image analysis was excellent (*k* = 0.8273 for SSFSE T2, *k* = 0.8143 for DWI and *k* = 0.8165 for ADC maps).

### Scanning time

Scanning time resulted significantly lower for Axial SSFSE T2 ARDL compared to NAÏVE with an average time of 19.08 ± 2.58 s compared with 24.11 ± 2.03 s (*P* < 0.001), with 31% of time reduction for ARDL protocol compared to NAÏVE protocol.

Similar results are appreciable for DWI sequence with a mean acquisition time of 207.33 (3.45 min) ± 54.03 s vs 513.60 (8.56 min) ± 98.69 s (*P* < 0.001), with 60% of time reduction for ARDL protocol compared to NAÏVE protocol.

## Discussion

The present study compared image quality of deep-learning MRI sequences with non-deep learning sequences. Results showed how SNR and CNR are higher for SSFSE T2 sequences of ARDL protocol compared to NAÏVE protocol including NON-DL dataset. No differences in SNR calculated from DWI between ARDL, NAÏVE and NON-DL dataset were observed as well as in CNR between ARDL and NAÏVE; on the other hand, CNR of DWI was higher in ARDL compared with NAÏVE protocol and in NAÏVE compared with NON-DL dataset. No differences in SNR and CNR for ADC maps were observed for all datasets.

Very positive results were also obtained for qualitative analysis that showed how ARDL protocol was superior to NAÏVE and NON-DL datasets for both SSFSE T2, DWI and ADC. In addition, acquisition time resulted significantly lower in ARDL protocol for both sequences tested.

Significant results in terms of quantitative and qualitative analysis for SSFSE T2 sequence are in line with data reported by Wang and colleagues [16] that reported how deep-learning sequences showed better image quality and fewer artifacts in prostate MRI compared with non-deep learning protocol.

Diffusion-weighted imaging analysis showed interesting results that need some consideration. DWI acquired without ARDL protocol was performed with high-quality parameters and it is possible to consider them as the ground truth. These acquisition parameters have returned a high-quality image with the disadvantages of a long acquisition time. In fact, quantitative analysis showed how there were no significant differences in terms of SNR and CNR with ARDL protocol acquired with a mean time of three minutes, 60% less than NAÏVE protocol. Moreover, the deep learning protocol showed improved image quality compared with the NAÏVE one. To the best of our knowledge, there are no studies performed on abdominal DWI; however, Misaka et al. [17] conducted an interesting study on female pelvis. They built a deep learning algorithm to improve single-shot turbo spin-echo (SSTSE) sequences and compare them with TSE, considered as ground truth, and SSTSE. Results showed how DL SSTSE were superior in terms of contrast ratio and SNR to SSTSE while no differences were observed with TSE. Image quality was higher for DL protocol compared to the others. Even though the sequences and the district are dissimilar, the approach of the analysis is comparable and in line with our results.

Similar results for DWI were obtained in other anatomical districts [18, 19]. An example is provided by Kaye and colleagues on prostate MRI [19]. They performed a comparison study on DWI obtained with a high average number with a retrospective guided denoising CNN that worked on low b values. Results performed on 118 patients divided into training and validation datasets of prostate MRI showed higher peak signal-to-noise ratio, higher structural similarity index and lower normalized mean square error compared to standard images. Also image quality resulted better for guided denoised CNN compared with the reference standard. In addition, the ADC map values obtained from denoised DW images had good agreement with the reference ADC values. Despite the retrospective method used and the different algorithm tested, our results are in line in terms of image quality and reduction of acquisition time and this aspect strength the role of AI and DL for clinical imaging. In fact, reduced acquisition time without affecting image quality would allow to improve clinical imaging scheduling.

Another aspect of the results obtained concerns the NON-DL dataset. In fact, in all the sequences analyzed, NON-DL dataset resulted inferior in terms of quantitative image analysis and image quality. Results are coherent with the actual meaning of the NON-DL dataset, which represents the acquisition obtained with non-optimized MRI parameters without the application of ARDL. NON-DL dataset is usually kept in the examinations for radiologists’ consultation. In fact, at the beginning radiologists might need some time to get confident with ARDL algorithm and the idea of non-DL images helps in that direction, even if they actually do use the ARDL dataset after a few scans.

The last aspect that needs important consideration is represented by the impact of ARDL on scanning time; it was faster for ARDL protocol compared with NAÏVE protocol, from 31% for SSFSE to 60% for DWI. Our results are in line with literature present with an average reduction of scanning time ranging from 30 to 60% [18, 20]. By reducing scanning time without affecting image quality represents an important goal achievable for AI. By so doing it would be possible to increase number of patients in the scheduling, without affecting the quality of examination, with a net improvement of healthcare process.

Despite the interesting results, our study has some limitations such as: lack of analysis of pathologic entities; only two sequences of the MRI upper abdomen protocol were tested also because ARDL for now has been approved for clinical use only for 2D sequences; lack of comparison with a standard NAÏVE protocol because we aimed to test a higher image quality protocol with DL to assess image quality; in fact, some acquisition parameters such as matrix or average for b values are different in SSFSE T2 and DWI acquisitions respectively.

## Conclusion

In conclusion, deep learning algorithm applied on MRI sequence acquisition for SSFSE T2 and DWI resulted better for image quality and comparable in terms of signal-to-noise ratio and contrast-to-noise ratio with high quality non deep learning protocol, by enabling a considerable reduction of acquisition time.
